# New homozygous *gpt* delta transgenic rat strain improves an efficiency of the *in vivo* mutagenicity assay

**DOI:** 10.1186/s41021-021-00195-1

**Published:** 2021-06-23

**Authors:** Kenichi Masumura, Tomoko Ando, Akiko Ukai, Sho Fujiwara, Shigeo Yokose, Xinyue You, Takayoshi Suzuki, Hiroyuki Hayashi, Takehiko Nohmi, Hisayoshi Takagi, Masamitsu Honma

**Affiliations:** 1 Division of Genetics and Mutagenesis, National Institute of Health Sciences, 3-25-26 Tonomachi, Kawasaki-ku, Kawasaki-shi, Kanagawa 210-9501 Japan; 2 Biotechnical Center, Japan SLC, Inc., 3-5-1, Aoihigashi, Naka-ku, Hamamatsu-shi, Shizuoka, 433-8114 Japan; 3 Division of Molecular Target and Gene Therapy Products, National Institute of Health Sciences, 3-25-26 Tonomachi, Kawasaki-ku, 210-9501 Kawasaki-shi, Kanagawa Japan; 4 School of Public Health, Hongqiao International Institute of Medicine, Shanghai Jiao Tong University School of Medicine, 227 South Chongqing Road, Shanghai, 200025 China; 5 Biologics Business Planning & Operation Dept, Meiji Seika Pharma Co., Ltd, 2-4-16 Kyobashi Chuo- ku, Tokyo, 104-8002 Japan; 6 Division of Pathology, National Institute of Health Sciences, 3-25-26 Tonomachi, Kawasaki-ku, Kawasaki-shi, Kanagawa 210-9501 Japan

**Keywords:** *gpt* delta transgenic rat, *gpt* assay, Spi^−^ assay, mutant frequency, mutation spectrum

## Abstract

**Background:**

Gene mutation assays in transgenic rodents are useful tools to investigate *in vivo* mutagenicity in a target tissue. Using a lambda EG10 transgene containing reporter genes, *gpt* delta transgenic mice and rats have been developed to detect point mutations and deletions. The transgene is integrated in the genome and can be rescued through an *in vitro* packaging reaction. However, the packaging efficiency is lower in *gpt* delta rats than in mice, because of the transgene in *gpt* delta rats being heterozygous and in low copy number. To improve the packaging efficiency, we herein describe a newly developed homozygous *gpt* delta rat strain.

**Results:**

The new *gpt* delta rat has a Wistar Hannover background and has been successfully maintained as homozygous for the transgene. The packaging efficiency in the liver was 4 to 8 times higher than that of existing heterozygous F344 *gpt* delta rats. The frequency of *gpt* point mutations significantly increased in the liver and bone marrow of *N*-nitroso-*N*-ethylurea (ENU)- and benzo[*a*]pyrene (BaP)-treated rats. Spi^−^ deletion frequencies significantly increased in the liver and bone marrow of BaP-treated rats but not in ENU-treated rats. Whole genome sequencing analysis identified ≥ 30 copies of lambda EG10 transgenes integrated in rat chromosome 1.

**Conclusions:**

The new homozygous *gpt* delta rat strain showed a higher packaging efficiency, and could be useful for *in vivo* gene mutation assays in rats.

**Supplementary Information:**

The online version contains supplementary material available at 10.1186/s41021-021-00195-1.

## Introduction

Genotoxicity is an important biomarker for carcinogenesis because many carcinogens are reactive to DNA and induce mutations in target organs [1]. To investigate *in vivo* mutagenicity in those organs/tissues, transgenic rodent gene mutation assays have been developed [2–4]. They use the lambda phage as a shuttle vector including reporter genes for mutations. Tandem copies of the lambda phage DNA, which is approximately 45–48 kb in size, are integrated in the host genome. The phages can be later recovered from the genomic DNA by *in vitro* packaging reactions and the mutated reporter genes phenotypically selected after introduction of the rescued phage to indicator *Escherichia coli* cells. The assays allow detection of *in vivo* mutations in any organs/tissues. In addition, DNA sequence analysis of the detected mutants can reveal the mutation spectra associated with chemical exposure. Transgenic rodent gene mutation assays have been adopted as OECD Test Guideline No. 488 and used for testing of genotoxicity of chemicals [3]. Muta Mice, BigBlue mice, and *gpt* delta mice are representative transgenic mice [5–7], while BigBlue rats and *gpt* delta rats were also developed [8–10]. Rats have been more frequently used for cancer bioassay and toxicological research for chemicals than mice. There are a number of chemicals that exhibit species differences in the carcinogenicity, and the target organs for cancer sometimes differ between species. Thus, genotoxicity assays using rats is important for examining the relationship between *in vivo* genotoxicity and carcinogenicity.

Specifically, *gpt* delta transgenic mice (C57BL/6J background) and rats (Sprague-Dawley and F344 background) use the lambda EG10 phage as a transgene [7, 8, 10]. Two distinct selection systems are available for *gpt* delta rodents. One is the 6-thioguanine (6TG) selection for point mutations in the *gpt* gene, and the other is Spi^−^ (sensitive to P2 interference) selection for deletions by simultaneous inactivation of the *gam* and *red* genes. Both *gpt* and Spi^−^ assays have been validated in both mice and rats with many chemical mutagens/carcinogens, UV, and ionizing radiations [11–13]. However, the rescue efficiency of the transgene from the host genome is much lower in *gpt* delta rats than in mice, because the transgene in *gpt* delta rats is heterozygous [8] and the number of copies integrated in the genome is lower, 14 copies maximum in rat chromosome 4 versus approximately 40 copies maximum per haploid in mouse chromosome 17 [14]. The lower packaging efficiency of *gpt* delta rats increases the assays’ cost by additional packaging reactions and plating. To overcome this disadvantage, we established new strain of homozygous *gpt* delta rat with a high EG10 copy number. Wistar Hannover (WH) rat is a well-characterized strain that has been used in general toxicity and carcinogenicity studies worldwide [15–18]. In this study, we show that the new homozygous *gpt* delta rats (WH background) have an improved packaging efficiency of transgene rescue. Further, the homozygous WH *gpt* delta rats showed mutagenic responses to benzo[*a*]pyrene (BaP) and *N*-nitroso-*N*-ethylurea (ENU). Genomic integration of the transgene was analyzed by whole genome sequencing (WGS). In short, the new homozygous *gpt* delta rat line could be useful to evaluate chemically-induced mutagenic and carcinogenic effects in rats.

## Materials and methods

### Establishment of homozygous ***gpt*** delta rats

Lambda EG10 DNA was purified from a liquid phage lysate with QIAGEN Lambda Maxi Kit (QIAGEN) and dissolved in 1/10-diluted TE buffer. All animal experiments in this study were performed by Japan SLC, Inc. (Shizuoka, Japan) and approved by the institutional animal care and use committee and followed recommendations for the handling, maintenance, treatment, and sacrificing of the animals. The lambda EG10 DNA solution was injected into the pronucleus of a fertilized zygote from WH rats. Healthy microinjected zygotes were promptly transplanted into the oviducts of female rats mated with sterilized male rats. The transplanted female rats yielded offspring. For weaning rats, presence of lambda EG10 DNA was checked by PCR using the primers described previously [7]. Transgene-positive founders were maintained and followed by brother-sister mating to generate a homozygous line. Transgene homozygosity was confirmed by progeny test and PCR.

### Estimation of packaging efficiency

Packaging efficiency was evaluated as described [19]. High molecular weight genomic DNA was extracted from the liver of *gpt* delta rat lines using RecoverEase DNA Isolation Kit (Agilent Technologies, Santa Clara, CA). Lambda EG10 phages were rescued by an *in vitro* packaging reaction using Transpack Packaging Extracts (Agilent Technologies). For each packaging reaction, 10 µL DNA was employed. *E. coli C* cells were infected with the packaged samples, mixed with molten soft agar, poured onto lambda agar plates, and incubated at 37 °C overnight. Phage plaques were counted and the number of rescued phages (plaque forming unit: p.f.u.) per packaging reaction was estimated. In addition, *E.coli* YG6020 cells were infected with the packaged phages, mixed with molten soft agar, poured onto M9 agar plates containing chloramphenicol (Cm), and incubated at 37 °C for 3 days. Cm-resistant colonies were counted and the number of rescued phages (colony forming units: c.f.u.) was estimated.

### Mutagenic treatment

Male and female WH homozygous *gpt* delta transgenic rats (line 2) were maintained and treated by Japan SLC, Inc. Administration started at 10 weeks of age. Five animals were used for each group. BaP (CAS No.: 50-32-8) and ENU (759-73-9) were purchased from Sigma Aldrich (MO, USA). Olive oil and saline were used as vehicle for BaP and ENU, respectively. BaP treatment: rats were treated with BaP (0, 62.5 and 125 mg/kg/day) by gavage for 28 days. Three days after the final treatment (day 31), tissue samples were collected and stored at − 80ºC (28 + 3d protocol recommended by OECD TG488). ENU treatment: rats were treated with ENU (50 mg/kg/day) intraperitoneally for 5 days. Twenty-six days after the final treatment (day 31), tissue samples were collected. Liver and bone marrow were used for gene mutation assays. Because the number of animals was limited, animal treatment was conducted in three separate experiments (Control and BaP-treated males, control and BaP-treated females, and ENU-treated males and females).

### Mutation assays and sequencing analysis

Genomic DNA was extracted from the liver and bone marrow of control and mutagen-treated rats, and then the transgene was recovered by *in vitro* packaging as described above. The *gpt* mutation assays were conducted as previously described [19]. For *gpt* assay, the rescued phages were incubated with *E.coli* YG6020 cells and poured onto M9 agar plates containing Cm and 6-thioguanine (6-TG). Infected cells were also poured onto agar plates containing Cm without 6-TG to determine the total number of rescued transgenes. The plates were then incubated at 37 °C for selection of 6-TG-resistant colonies, and the *gpt* mutant frequency (MF) calculated by dividing the number of *gpt* mutants by the number of rescued transgenes. A DNA fragment containing the 456-bp coding region for the *gpt* gene was amplified by colony-direct PCR and *gpt* mutations were characterized by DNA sequencing with a sequencing primer gptA2 (5′-TCTCGCGCAACCTATTTTCCC-3′). The specific *gpt* mutation frequency of each type of mutation was calculated by *gpt* MF × proportion of the type of sequenced independent mutations.

The Spi^−^ mutation assay was conducted as previously described [19] with modification. Rescued phages were incubated with *E.coli* XL-1 Blue MRA P2 cells and poured onto lambda-trypticase agar plates and then incubated at 37 °C to detect Spi^−^ mutant plaques. The phages were also incubated with *E.coli* XL-1 Blue MRA and poured onto agar plates to determine the total number of rescued phages. To confirm the Spi^−^ phenotype, Spi^−^ candidates were spotted on three types of plates on which XL-1 Blue MRA, XL-1 Blue MRA P2, or WL95 P2 strains were spread with soft agar. True Spi^−^ mutants, able to create plaques on all plates, were counted. The Spi^−^ MF was calculated by dividing the number of Spi^−^ mutants by the number of rescued phages. Spi^−^ mutant phage lysates were obtained by infecting *E. coli* LE392 with the Spi^−^ mutants. Lambda EG10 DNA was extracted from liquid phage lysates by Puregene Kit (QIAGEN) and used for PCR and sequencing analysis to determine the deleted regions. The specific Spi^−^ mutation frequency of each type of mutations was calculated by Spi^−^ MF x proportion of the types of mutations.

### Detection of transgene integration site in the rat genome

Genomic DNA was extracted from the liver of homozygous *gpt* delta rats (line 2) using a DNeasy Kit (QIAGEN). WGS analysis using NovaSeq6000 (Illumina, CA, USA) and insert prediction were performed by Macrogen Japan Corp. (Tokyo, Japan). More than 90 Gb of sequence data (> 30 read depth in average) was obtained by 150 bp paired-end sequencing and mapped onto reference sequences using Burrows-Wheeler Aligner (BWA). The reference sequences used in the analyses were the rat genome (UCSC. RGSC 6.0/rn6) and lambda EG10 sequence [14]. Predicted junctions between rat chromosome and lambda EG10 sequences were confirmed by PCR and Sanger sequencing. The transgene integration site was independently confirmed by MinION long-lead DNA sequencer (Oxford Nanopore technologies, UK). PCR primers were designed for genotyping of the homozygous *gpt* delta rat (Supplementary Fig. 1).

### Analysis of transgene copy number

The copy number of transgenes integrated into rat genomes was estimated as previously reported [14]. Data analysis was performed by GeneBay Inc. (Kanagawa, Japan). Briefly, the WGS data was mapped onto reference sequences using BWA. Pair-reads for which one read was mapped on the transgene, and the other on the rat genome sequence were counted. The number of pair-reads covering upstream or downstream junctions was divided by two to estimate the average number of pair-reads covering a single unique site in the genome. Next, the number of pair-reads for which two reads were mapped on both left and right transgene arms, indicating that pair-reads covered the junction of two transgene copies, was counted. Then, the number of pair-reads covering the junction of two transgene copies was divided by the number of pair-reads covering a single unique site in the genome. The calculated multiplicity indicates the number of junctions of two transgene copies. Transgene copy number was estimated from the number of junctions. In addition, the integration pattern of multiple transgene copies was analyzed using mapped reads data. Position of pair-reads was mapped on the lambda EG10 sequence and plotted on a graph.

### Statistical analysis

MFs in each dose group are presented with standard deviation (SD). Comparison of MFs between mutagen-treated groups versus vehicle control was analyzed by Dunnett’s test or Steel test. Comparison between male and female was analyzed by Student’s or Welch’s t-test. Comparisons of mutation spectrum were analyzed by Fisher’s exact test and chi-square test.

## Results

### Development of the homozygous ***gpt*** delta rat

**Fig. 1:**
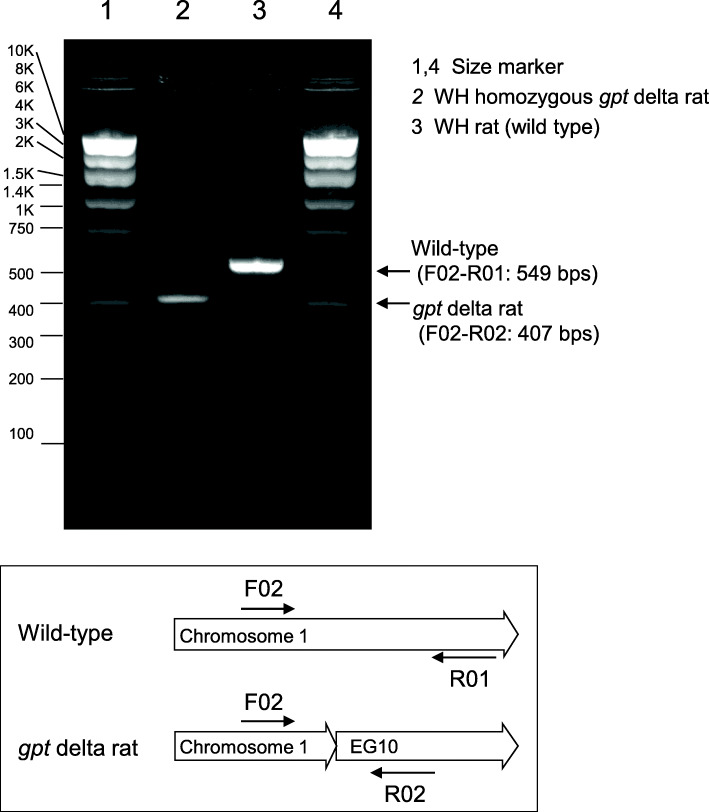
Genomic integration and homozygosity of EG10 transgene. Genomic integration of transgene was confirmed by multiplex PCR using three primers. Primer sequences are presented in Supplementary Fig. 1. Genomic DNA extracted from liver of WH homozygous *gpt* delta rat line 2 and wild-type rat was used as a PCR template. A diagram shows primer positions for each genotype.

The lambda EG10 DNA was injected into a fertilized zygote prepared from WH rats and transplanted into female rats yielding offspring. Transgenic rat candidates were checked for genomic lambda EG10 DNA presence by PCR. Transgene recovery was confirmed by *in vitro* packaging. Transgene-positive founders were maintained by brother-sister mating. Transgene homozygosity was confirmed by progeny test and PCR. Two candidate WH homozygous *gpt* delta rat lines were obtained. No adverse phenotype was observed while breeding for these lines. For one candidate line (line 2), genomic integration site of transgene was analyzed by WGS. Insert prediction identified that multiple copies of the lambda EG10 sequence are integrated in a single site of rat chromosome 1. Integration site and homozygosity of the transgene were confirmed by PCR (Fig. 1 and Supplementary Fig. 1).

### Packaging efficiency of homozygous ***gpt*** delta rat candidates

**Fig. 2:**
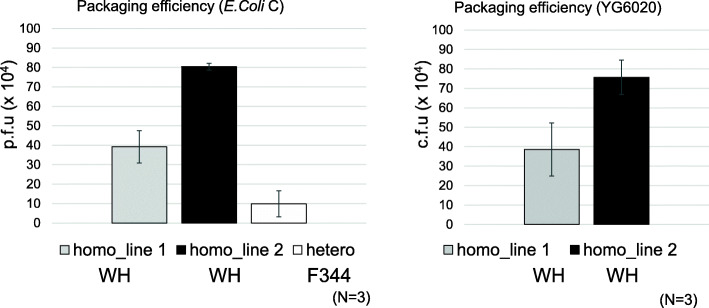
Packaging efficiencies of homozygous *gpt* delta rats. Two candidate WH homozygous *gpt* delta rat lines were analyzed for packaging efficiency. F344 heterozygous *gpt* delta rats were used as a control. Three animals were used in each group. Genomic DNA was extracted from the liver of untreated rats. Three independent *in vitro* packaging reactions using 10 µL DNA were conducted for each DNA sample. *E. coli* C and YG6020 cells were infected with the packaged phages. The number of rescued phages per packaging reaction (plaque forming unit: p.f.u or colony forming unit: c.f.u) was estimated. Error bar represents standard deviation

Two candidate homozygous *gpt* delta rat lines were analyzed for packaging efficiency. Existing heterozygous F344 *gpt* delta rats were used as control. Genomic DNA was extracted from the liver (80–100 mg) of untreated rats. For each DNA sample, three independent *in vitro* packaging reactions were conducted using 10 µL DNA. Both WH homozygous *gpt* delta rat lines showed that a packaging efficiency of liver DNA around 4 × 10^5^ (line 1) and 8 × 10^5^ (line 2) p.f.u/reaction (Fig. 2), with the packaging efficiency of line 2 being approximately 8 times higher than that of heterozygous F344 *gpt* delta rats. The p.f.u. for *E. coli* C and c.f.u. for YG6020 were similar. From this point onwards, we decided to use line 2 for mutagenesis experiments because higher packaging efficiency is preferred.

### Mutant frequencies of BaP- and ENU-treated homozygous ***gpt*** delta rats

Line 2 homozygous *gpt* delta rats were treated with BaP and ENU. Suppression of body weight gain was observed in a 62.5 mg/kg/day BaP-treated male and female and in a 125 mg/kg/day BaP-treated female, while slight body weight loss was observed in a 125 mg/kg/day BaP-treated male at the end of treatment. Similarly, suppression of body weight gain was observed in a 50 mg/kg/day ENU-treated male and female. Tissue samples were collected at day 31 (28 + 3d for BaP and 5 + 26d for ENU). Genomic DNA was extracted from the liver and bone marrow and a *gpt* assay for point mutations and Spi^−^ assay for deletions were performed.

**Fig. 3:**
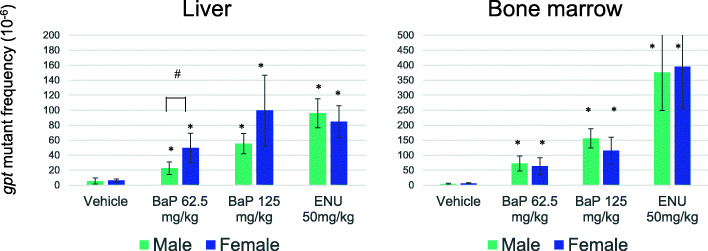
The *gpt* mutant frequencies in the liver and bone marrow of BaP- or ENU-treated homozygous *gpt* delta rats. Error bar represents standard deviation. * *P* < 0.05, significantly different from vehicle control (Steel test). # *P* < 0.05, significantly different between male and female (t-test)

The result of the *gpt* assay is shown in Fig. 3 (and Supplementary Table S1 and S2). The spontaneous *gpt* MFs in the liver of homozygous WH rats were 5.3 ± 4.0 × 10^− 6^ in male and 6.5 ± 1.9 × 10^− 6^ in female and those in bone marrow were 4.3 ± 2.2 × 10^− 6^ in male and 6.3 ± 1.3 × 10^− 6^ in female. The *gpt* MFs significantly increased in the liver and bone marrow of BaP-treated rats in a dose-dependent manner, so did those in the ENU-treated groups which significantly increased in both tissues. The *gpt* MFs in the liver of BaP-treated females were slightly higher than those of males, but significantly different only in the 62.5 mg/kg/day group (*P* < 0.05, t-test). No significant sex difference was observed in the bone marrow of BaP- and ENU-treated groups or in the spontaneous and mutagen-induced mutation spectra in the liver (Table 1). No clonal germline mutation in the *gpt* gene was observed. In untreated rats, G:C to A:T transitions, G:C to T:A transversions and small deletions (< 100 bp) were predominantly detected. Sixty % (6 of 10 in sum of male and female) of 1 bp deletions occurred at G:C bps. In the BaP-treated group, G:C to T:A transversions were predominant, followed by other base substitutions at G:C bps. Pooling data from male and female rats, a significant increase of G:C to T:A and G:C to C:G transversions in the BaP-treated group was observed (*P* < 0.01, Fisher’s exact test). Eighty-three  % (5 of 6 in sum of male and female) of 1 bp deletions occurred at G:C bps. In the ENU-treated group, base substitutions at A:T bps and G:C to A:T transitions were predominant, and a significant increase of A:T to T:A and A:T to C:G was observed (*P* < 0.01). Moreover, two of three 1 bp deletions occurred at A:T bps. Hotspots of *gpt* mutations, which were defined in this study as those detected from at least 3 rats among 10 (5 males and 5 females), were G:C to A:T at nucleotide position 64, 110 and 115 in control group, G:C to T:A at 59, 115, 401 and 402 in BaP-treated group, and A:T to G:C at 263 and A:T to T:A at 2, 263 and 415 in ENU-treated group.

**Table 1:** The *gpt* mutation spectra in the liver of BaP and ENU-treated homozygous *gpt* delta rats

	0 mg/kg/day (28 + 3d)						BaP 125 mg/kg/day (28 + 3d)						ENU 50 mg/kg/day (5 + 26d)					
	male			female			male			female			male			female		
	No.	%	MF (x10^− 6^)	No.	%	MF (x10^− 6^)	No.	%	MF (x10^− 6^)	No.	%	MF (x10^− 6^)	No.	%	MF (x10^− 6^)	No.	%	MF (x10^− 6^)
Base substitution																		
Transition																		
G:C to A:T	15	40.5	2.2	17	45.9	3.0	5	12.2	6.8	7	16.3	16.2	7	15.6	14.9	9	21.4	18.1
(at CpG)	(6)			(10)			(3)			(1)			(1)			(2)		
A:T to G:C	4	10.8	0.6	2	5.4	0.3	2	4.9	2.7	0	0.0	0.0	10	22.2	21.3	5	11.9	10.1
Transversion																		
G:C to T:A	6	16.2	0.9	8	21.6	1.4	19	46.3	25.7	15	34.9	34.8	7	15.6	14.9	4	9.5	8.1
G:C to C:G	0	0.0	0.0	3	8.1	0.5	6	14.6	8.1	9	20.9	20.9	0	0.0	0.0	1	2.4	2.0
A:T to T:A	0	0.0	0.0	1	2.7	0.2	0	0.0	0.0	2	4.7	4.6	11	24.4	23.4	14	33.3	28.2
A:T to C:G	1	2.7	0.1	0	0.0	0.0	0	0.0	0.0	2	4.7	4.6	7	15.6	14.9	7	16.7	14.1
Deletion	8	21.6	1.2	5	13.5	0.9	6	14.6	8.1	5	11.6	11.6	3	6.7	6.4	1	2.4	2.0
1 bp	6			4			3			3			2			1		
> 2 bps	2			1			3			2			1			0		
Insertion	2	5.4	0.3	0	0.0	0.0	2	4.9	2.7	1	2.3	2.3	0	0.0	0.0	1	2.4	2.0
Others	1	2.7	0.1	1	2.7	0.2	1	2.4	1.4	2	4.7	4.6	0	0.0	0.0	0	0.0	0.0
Total	37	100	5.3	37	100	6.5	41	100	55.4	43	100	99.7	45	100	95.9	42	100	84.7

**Fig. 4:**
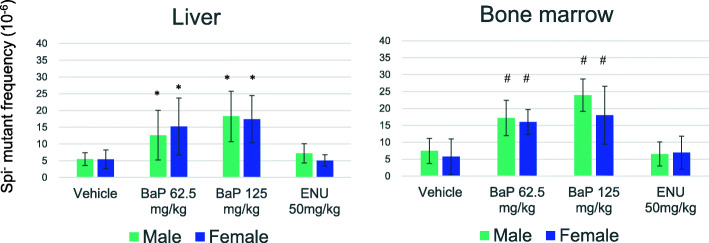
The Spi^−^ mutant frequencies in the liver and bone marrow of BaP- or ENU- treated homozygous *gpt* delta rats. Error bar represents standard deviation. * *P* < 0.05, significantly different from vehicle control (Steel test). # *P* < 0.05, significantly different from vehicle control (Dunnett’s test)

The MF results of Spi^−^ deletions are shown in Fig. 4 (and Supplementary Table S3 and S4). The spontaneous Spi^−^ MFs in the liver of homozygous WH rats were 5.5 ± 1.9 × 10^− 6^ in male and 5.4 ± 2.8 × 10^− 6^ in female and those in bone marrow were 7.5 ± 3.7 × 10^− 6^ in male and 5.8 ± 5.2 × 10^− 6^ in female. The Spi^−^ MFs significantly increased in the liver and bone marrow of BaP-treated rats. On the other hand, the Spi^−^ MFs in ENU-treated groups were similar to those of controls. No sex differences were observed in any dose groups. The Spi^−^ mutation spectra in the liver are shown in Table 2 (and Supplementary Table S5). No sex differences were observed in the spontaneous and mutagen-treated groups. In the control group, 1 bp deletions at repetitive sequences were predominantly observed. In the BaP-treated group, after pooling male and female data, a significant difference from controls was observed in the mutation spectrum (*P* < 0.01, chi-square test). The increase of 1 bp deletions at G:C sequences mainly contributed to the higher Spi^−^ MF in the BaP-treated group. On the other hand, 1 bp deletions at A:T sequences and large deletions > 1 kb in size were not clearly induced in the BaP-treated group. In the ENU-treated group, no significant difference from control was observed in the mutation spectra.

**Table 2:** The Spi^−^ mutation spectra in the liver of BaP and ENU-treated homozygous *gpt* delta rats

	0 mg/kg/day (28 + 3d)						BaP 125 mg/kg/day (28 + 3d)						ENU 50 mg/kg/day (5 + 26d)					
	male			female			male			female			male			female		
	No.	(%)	MF (x10^− 6^)	No.	(%)	MF (x10^− 6^)	No.	(%)	MF (x10^− 6^)	No.	(%)	MF (x10^− 6^)	No.	(%)	MF (x10^− 6^)	No.	(%)	MF (x10^− 6^)
One bp deletion	34	85.0	4.6	34	77.3	4.2	20	71.4	13.0	18	81.8	14.2	11	64.7	4.7	13	76.5	3.9
at G:C	19	47.5	2.6	12	27.3	1.5	14	50.0	9.1	15	68.2	11.9	7	41.2	3.0	5	29.4	1.5
at A:T	15	37.5	2.0	22	50.0	2.7	6	21.4	3.9	3	13.6	2.4	4	23.5	1.7	8	47.1	2.4
> 2 bps deletion	5	12.5	0.7	7	15.9	0.9	4	14.3	2.6	1	4.5	0.8	2	11.8	0.8	3	17.6	0.9
Base substitution	0	0.0	0.0	1	2.3	0.1	3	10.7	2.0	2	9.1	1.6	3	17.6	1.3	0	0.0	0.0
Insertion	0	0.0	0.0	0	0.0	0.0	1	3.6	0.7	0	0.0	0.0	0	0.0	0.0	0	0.0	0.0
Complex	1	2.5	0.1	2	4.5	0.2	0	0.0	0.0	1	4.5	0.8	1	5.9	0.4	1	5.9	0.3
Total	40	100.0	5.5	44	100.0	5.4	28	100.0	18.3	22	100.0	17.4	17	100.0	7.2	17	100.0	5.1

### Transgene integration in the rat genome

**Fig. 5:**
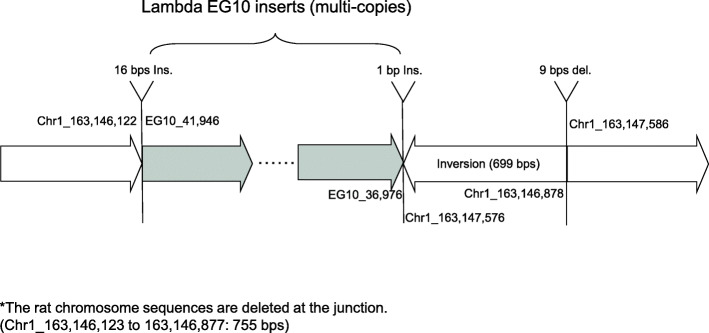
Integration of lambda EG10 transgenes in chromosome 1 of homozygous *gpt* delta rats. A diagram of the insertion region of EG10 sequences in the *gpt* delta rat genome is shown. The dark arrow represents the lambda EG10 transgene copies, and the white arrow represents the rat chromosome. Deletion (755 bps), inversion (699 bps) and small Indels (16 bps and 1 bp insertions, 9 bps deletion) in the rat genome were detected at the junction

Transgene genomic integration was analyzed by WGS. The inserted junction of the WH homozygous *gpt* delta rat (line 2) is shown in Fig. 5. Multiple copies of the lambda EG10 sequence are integrated in a single site of rat chromosome 1. At the junction, a 755-bps deletion of rat genome sequence was detected. Inserted position is in intron between exon 1 and exon 2 of the *B3gnt6* gene coding UDP-GlcNAc:betaGal beta-1,3-N-acetylglucosaminyltransferase 6, suggesting a loss of the gene function. At the upstream junction, a 16 bps insertion was detected. At the downstream junction, a 1 bp insertion, a 699 bps inverted sequence and a 9 bps deletion were detected.

EG10 copy number was calculated as described in Methods. The numbers of sequenced read-pairs covering the junction between the transgene and rat chromosome were 50 upstream and 43 downstream. Therefore, the number of read-pairs covering a single unique site in the genome could be estimated as (50 + 43)/2 = 46.5. The number of read-pairs covering the junction between two transgene copies in a head-to-tail direction was 2130. Thus, the number of normal head-to-tail junctions was estimated as 2130/46.5 = 46 junctions. No head-to-head or tail-to-tail junction was detected. The number of read-pairs for abnormal junctions with transgene rearrangement was 629. Therefore, the number of abnormal junctions was estimated as 629/46.5 = 14. Those abnormal junctions were visualized by a distribution map of read-pairs (Supplementary Fig. 2), which confirmed the 14 abnormal (rearranged and/or fragmented) junctions. In total, the inserted transgene copies contain 45 normal head-to-tail junctions and 14 abnormally rearranged junctions between upstream and downstream junctions to the rat’s genome. Because the order of each junction is unknown, the number of intact (functional) EG10 sequence copies could be estimated between a 30 ~ 44 per haploid.

## Discussion

Although originally developed as Sprague-Dawley rats, *gpt* delta transgenic rats were later backcrossed to generate F344 rats [8, 10]. However, these rats could not be bred in homozygosity because pups could not survive due to teeth deficiencies [8]. An issue associated with the heterozygosity of the original *gpt* delta rats was the limited transgene number and the lower rescue efficiency in *in vitro* packaging. In this study, a new line of *gpt* delta transgenic rats was generated by lambda EG10 DNA microinjection into fertilized WH rat eggs. WH rat is a widely used strain in toxicity and carcinogenicity studies as well as F344 and Sprague-Dawley rats. Two candidate homozygous *gpt* delta rat lines were obtained and successfully maintained to improve the rescue efficiency for efficient mutation assays. The packaging efficiency of homozygous *gpt* delta rats was 4 to 8 times higher than that of the original heterozygous F344 *gpt* delta rats (Fig. 2). The result suggests that the higher rescue efficiency is caused not only by transgene homozygosity but also because of the higher number of transgene copies integrated per haploid genome.

The spontaneous *gpt* MFs in the liver and bone marrow of homozygous WH rats were in a similar range to those in the tissues of heterozygous F344 rats (2 to 8 × 10^− 6^) [10, 20, 21]. In BaP-treated rats, the *gpt* MFs significantly increased in the liver and bone marrow with a dose dependency (Fig. 3). The BaP-induced *gpt* MFs were comparable with those of previously reported BaP-treated heterozygous male F344 *gpt* delta rats under the same 28-day dosing design [22], while the *gpt* MF in the liver of BaP-treated female was slightly higher than in males, it was significant only in the 62.5 mg/kg/day group. On the other hand, there was no sex difference in *gpt* MFs in the bone marrow. However, it should be noted that BaP dosing was performed in independent experiments for males and females (See Materials and Methods). Therefore, the sex difference in the BaP mutagenic sensitivity is not clear in the present study. In the ENU-treated groups, the *gpt* MFs significantly increased in both tissues without sex differences. The ENU-induced *gpt* MF was higher in the bone marrow than in the liver, suggesting that the bone marrow, a tissue of rapid cell-proliferation, could be more sensitive to direct alkylating mutagens such as ENU.

With respect to spontaneous *gpt* mutations in liver, G:C to A:T transitions, G:C to T:A transversions and small deletions were predominantly observed (Table 1), which are known as common characteristics of the spontaneous *gpt* mutations detected in many tissues in both rats and mice [10, 23, 24]. Hotspots of spontaneous *gpt* mutations were commonly observed as G:C to A:T transitions at 5’-CpG-3’ sites, namely at position 64, 110 and 115 in the *gpt* gene [24]. In the BaP-treated group, G:C to T:A transversions were predominantly induced, followed by other base substitutions at G:C bps. BaP induces *N*^2^-guanine DNA adducts after metabolic activation, inducing mainly G:C to T:A transversions and other point mutations at G:C bps [25, 26]. The same mutation spectrum characteristics were observed in the colon of BaP-treated *gpt* delta mice [27]. In the ENU-treated group, base substitutions at A:T bps and G:C to A:T transitions were predominantly induced. ENU generates a variety of DNA adducts, in which *O*^4^-ethylthymine, *O*^2^-ethylthymine, and *O*^6^-ethylguanine are considered responsible for causing A:T to G:C, A:T to T:A, and G:C to A:T mutations, respectively [28–33]. These common characteristic ENU-induced mutations were reported in various transgenic mice gene mutation assay models using *gpt*, *lacZ* and *lacI* as reporter genes [34–38]. No significant sex difference was observed neither in the spontaneous nor in the mutagen-induced mutation spectra. No germline mutation in the *gpt* gene was observed in the homozygous WH *gpt* delta rats. One germline base substitution, A:T to T:A at a position 299 in the *gpt* gene, is unintentionally present in a copy of transgene in the original heterozygous *gpt* delta rats [14]. Although this mutation does not affect mutation assay, it should be excluded in the analysis of mutation spectrum.

The spontaneous Spi^−^ MFs in the liver and bone marrow of homozygous WH rats were comparable to those in the liver of heterozygous Sprague-Dawley and F344 *gpt* delta rats (2.8 to 4.4 × 10^− 6^) [11]. In contrast to *gpt* MFs, the Spi^−^ MFs significantly increased in the liver and bone marrow of BaP-treated rats, but not in ENU-treated rats. The Spi^−^ assay is a selection system that preferentially detects deletions including 1 bp frameshift mutations [7, 39]. Since ENU predominantly induces base substitutions, it enhanced *gpt* MF but not Spi^−^ MF. Our result is consistent with the observation in heterozygous *gpt* delta rats [8]. Further, we observed no significant sex difference in any dose groups.

With respect to spontaneous Spi^−^ mutations in the liver, 1 bp deletions at repetitive base were predominantly observed. Hot spots of spontaneous Spi^−^ mutations were commonly observed as 1 bp deletions at 227–231 (5ʹ-AAAAA-3ʹ), at 286–289 (5ʹ-GGGG-3ʹ) and at 295–300 (5ʹ-AAAAAA-3ʹ) in the *gam* gene [20, 39]. *gam* and *redBA* gene translation is probably linked. Therefore, 1 bp deletions in the *gam* gene may interfere with the beginning of translation of downstream *redBA* genes, functionally inactivating not only *gam* but also *redBA*, which could be the reason why 1 bp deletions in the *gam* gene could induce Spi^−^ mutations [39, 40]. In the BaP-treated group, the increase in 1 bp deletions at G:C sequences contributed to the higher Spi^−^ MF (Table 2). It is reasonable that BaP induces guanine adducts in the DNA and repair or replication errors cause base substitutions but also frameshifts at G:C bp. Therefore, the higher percentage of 1 bp deletions at G:C bps observed in the BaP-treated group may be consistent with *gpt* mutations. In the ENU-treated group, no significant difference in Spi^−^ MF and mutation spectra from control was observed. It should be noted that a few base substitutions at G:C bp in the BaP-treated group and at A:T bp in the ENU-treated group were detected. Those may be induced by BaP-induced guanine adducts and ENU-induced adenine adducts, respectively. Base substitutions rarely cause Spi^−^ mutation because inactivation of both *gam* and *redBA* genes are usually induced by deletions in that region. Some mutants with base substitutions could be the result of a leaky phenotype of the P2 lysogen used in Spi^−^ selection [40]. Those base substitutions in the *gam* gene might be detected when the mutagenic treatment strongly induced base substitutions [20].

The genomic integration site of the lambda EG10 transgene was identified at a single site of rat chromosome 1 in homozygous *gpt* delta rats (Fig. 5). The insertion is located at the intron between exon 1 and exon 2 of the *B3gnt6* gene. B3GNT6 is involved in the synthesis of core 3 O-linked carbohydrate structures on mucin-type glycoproteins [41]. Transgene integration suggests a functional loss of this gene, since the lambda EG10 DNA is about 48 kb in size and > 40 copies are inserted in tandem at the single site, and an insertion of about 2 Mb of exogeneous sequence in a chromosome may affect the functions of other genes in surrounding regions. On the other hand, no adverse phenotype was observed while breeding this line of homozygous WH *gpt* delta rats. In available transgenic rodent models breeding for ≥ 20 years, a significant genotypic change such as transgene loss or genomic rearrangements has not been reported [2]. The transgenes are heavily methylated and not expressed in the host genome, suggesting that they could be stable and out of selection bias [42, 43]. The genomic integration profile of the transgene was analyzed by WGS. It suggested that there are 45 normal head-to-tail junctions and 14 abnormal rearranged junctions between EG10 copies, leading to an estimation of ≥ 30 intact copies per haploid. In the heterozygous F344 *gpt* delta rat genome, the inserted transgenes reportedly contain 15 head-to-tail junctions and 2 abnormal junctions [14]. Therefore, the homozygous WH *gpt* delta rat may contain 2 to 3-fold more transgene copies per haploid than the heterozygous *gpt* delta rat, and a comparable number to the homozygous C57BL/6J *gpt* delta mouse (41 head-to-tail junctions and 16 abnormal junctions per haploid) [14]. Together, the analyses support that the homozygous WH *gpt* delta rat could achieve higher EG10 vector rescue efficiency from genomic DNA than heterogyzous F344 *gpt* delta rat.

## Conclusions

The newly established homozygous WH *gpt* delta rats could considerably improve the lambda EG10 transgene rescue efficiency and be useful to detect *in vivo* mutagenicity (*gpt* point mutations and Spi^−^ deletions) in rats. The new homozygous WH *gpt* delta rats showed similar sensitivity and mutation spectra to those of heterozygous F344 *gpt* delta rats when they were treated with BaP and ENU. Further, WGS analyses showed that transgenes are integrated in a single position in chromosome 1, including ≥ 30 intact copies per haploid. Those results suggested that the homozygous WH *gpt* delta rats could be useful for mutagenicity test of chemicals. WH rats are used not only for carcinogenicity studies but also for general toxicity studies. Further studies will be necessary to validate the usefulness of homozygous WH *gpt* delta rats for integration of *in vivo* genotoxicity into general toxicity assays.

## Supplementary information

Additional file 1: **Supplementary Fig. 1.** Homozygous *gpt* delta rat genotyping. **Supplementary Fig. 2.** Distribution map of read-pairs covering EG10 copy junctions in homozygous *gpt* delta rats.

**Additional file 2.** Supplementary Table S1-5.

## Data Availability

All data generated or analyzed during this study are included in this published article and its supplementary information files.

## Abbreviations

6TG: 6-thioguanine
BaP: benzo[*a*]pyrene
BWA: Burrows-Wheeler Aligner
c.f.u.: colony forming unit
Cm: chloramphenicol
ENU: *N*-ethyl-*N*-nitrosourea
MF: mutation frequency
p.f.u.: plaque forming unit
SD: standard deviation
TGR: transgenic rodent gene mutation assay
WGS: whole genome sequencing
WH: Wistar Hannover
