# Supplementary material for: New homozygous gpt delta transgenic rat strain improves an efficiency of the in vivo mutagenicity assay
Source: Genes Environ. 2021 Jun 23;43:25. doi: 10.1186/s41021-021-00195-1 (PMC8220708; doi:10.1186/s41021-021-00195-1)
Supplement: Supplementary file 1 — Supplementary Fig. 1. Homozygous gpt delta rat genotyping. Supplementary Fig. 2. Distribution map of read-pairs covering EG10 copy junctions in homozygous gpt delta rats. [file 41021_2021_195_MOESM1_ESM.pptx]

## Slide 1
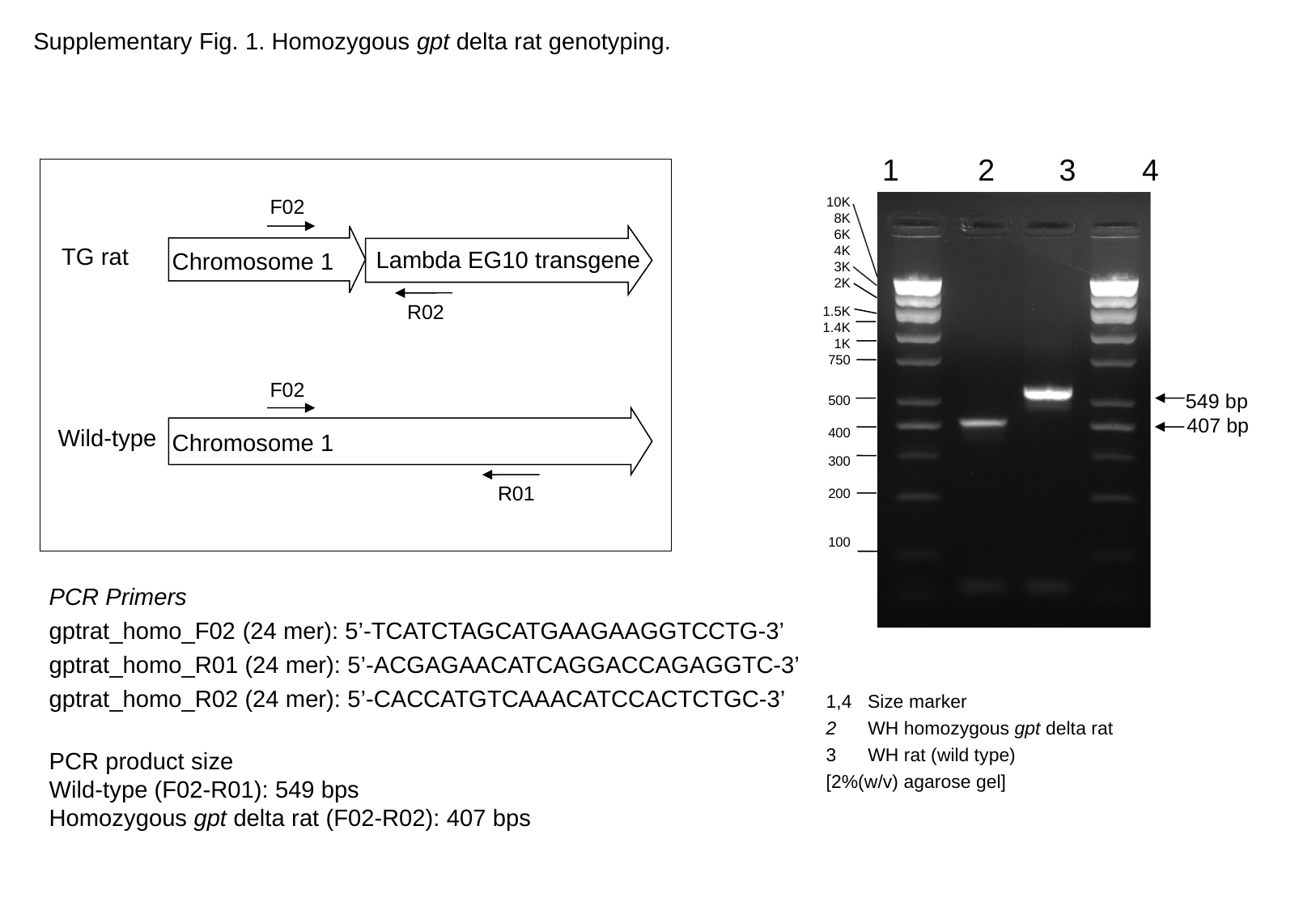

Supplementary Fig. 1. Homozygous gpt delta rat genotyping.
1 　2 　3 　4
F02
10K
8K
6K
4K
3K
2K
1.5K
1.4K
1K
750
500
400
300
200
100
TG rat
Lambda EG10 transgene
Chromosome 1
R02
F02
549 bp
407 bp
Wild-type
Chromosome 1
R01
PCR Primers
gptrat_homo_F02 (24 mer): 5’-TCATCTAGCATGAAGAAGGTCCTG-3’
gptrat_homo_R01 (24 mer): 5’-ACGAGAACATCAGGACCAGAGGTC-3’
gptrat_homo_R02 (24 mer): 5’-CACCATGTCAAACATCCACTCTGC-3’
PCR product size
Wild-type (F02-R01): 549 bps
Homozygous gpt delta rat (F02-R02): 407 bps
1,4 Size marker
2 WH homozygous gpt delta rat
3 WH rat (wild type)
[2%(w/v) agarose gel]

## Slide 2
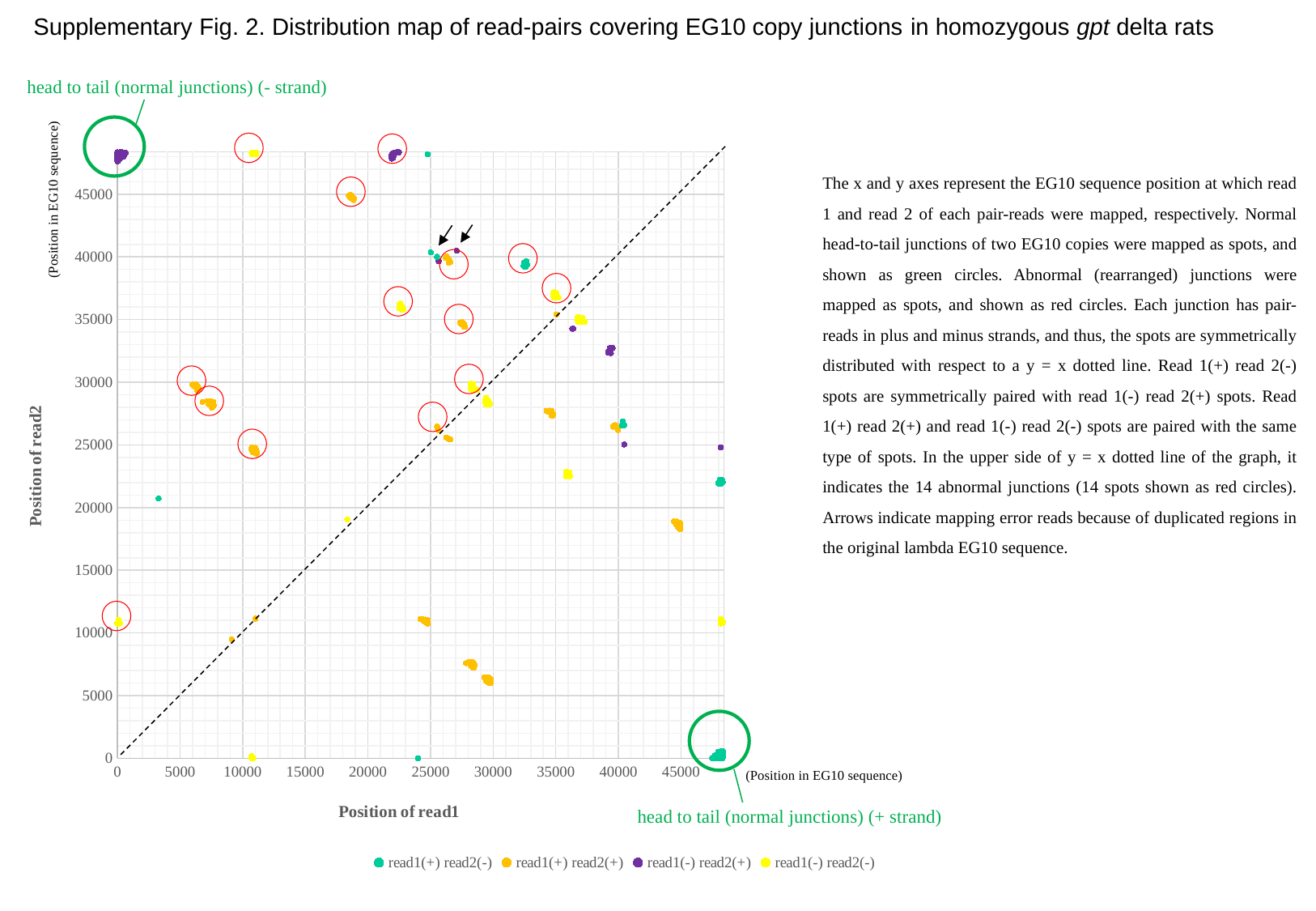

Supplementary Fig. 2. Distribution map of read-pairs covering EG10 copy junctions in homozygous gpt delta rats
head to tail (normal junctions) (- strand)
### Chart
| Category | | | | |
|---|---|---|---|---|
The x and y axes represent the EG10 sequence position at which read 1 and read 2 of each pair-reads were mapped, respectively. Normal head-to-tail junctions of two EG10 copies were mapped as spots, and shown as green circles. Abnormal (rearranged) junctions were mapped as spots, and shown as red circles. Each junction has pair-reads in plus and minus strands, and thus, the spots are symmetrically distributed with respect to a y = x dotted line. Read 1(+) read 2(-) spots are symmetrically paired with read 1(-) read 2(+) spots. Read 1(+) read 2(+) and read 1(-) read 2(-) spots are paired with the same type of spots. In the upper side of y = x dotted line of the graph, it indicates the 14 abnormal junctions (14 spots shown as red circles). Arrows indicate mapping error reads because of duplicated regions in the original lambda EG10 sequence.
(Position in EG10 sequence)
(Position in EG10 sequence)
head to tail (normal junctions) (+ strand)
